# Analysis and Comparison of the Antioxidant Component of *Portulaca Oleracea* Leaves Obtained by Different Solid-Liquid Extraction Techniques

**DOI:** 10.3390/antiox6030064

**Published:** 2017-08-12

**Authors:** Monica Gallo, Esterina Conte, Daniele Naviglio

**Affiliations:** 1 Department of Molecular Medicine and Medical Biotechnology, University of Naples Federico II, via Pansini, 5, Naples 80131, Italy; 2 Department of Chemical Sciences, University of Naples Federico II, via Cintia, Naples 80126, Italy; esterconte@hotmail.it (E.C.); naviglio@unina.it (D.N.)

**Keywords:** antioxidants, polyphenols, solid-liquid extraction techniques, DPPH assay, HPLC

## Abstract

*Portulaca oleracea* is a wild plant pest of orchards and gardens, but is also an edible vegetable rich in beneficial nutrients. It possesses many antioxidant properties due to the high content of vitamins, minerals, omega-3 essential fatty acids and other healthful compounds; therefore, the intake of purslane and/or its bioactive compounds could help to improve the health and function of the whole human organism. Accordingly, in this work it was analyzed and compared to the extractive capacity of the antioxidant component of purslane leaves obtained by solid-liquid extraction techniques such as: hot-maceration, maceration with ultrasound, rapid solid-liquid dynamic extraction using the Naviglio extractor, and a combination of two techniques (mix extraction). The chromatographic analysis by High Performance Liquid Chromatography (HPLC) of the methanolic extract of dried purslane leaves allowed the identification of various polyphenolic compounds for comparison with the standards. In addition, the properties of the different extracts were calculated on dry matter and the antioxidant properties of the total polyphenol components analyzed by the DPPH (2,2-diphenyl-1-picrylhydrazyl) assay. The results showed that mix extraction was the most efficient compared to other techniques. In fact, it obtained a quantity of polyphenols amounting to 237.8 mg Gallic Acid Equivalents (GAE)/100 g of fresh weight, while in other techniques, the range varied from 60–160 mg GAE/100 g fresh weight. In addition, a qualitative analysis by Liquid Chromatography-Tandem Mass Spectrometry (LC/MS/MS) of the phenolic compounds present in the purslane leaves examined was carried out. The compounds were identified by comparison of their molecular weight, fragmentation pattern and retention time with those of standards, using the “Multiple Reaction Monitoring” mode (MRM). Therefore, this study allowed the re-evaluation of a little-known plant that possesses as its beneficial properties, a great potential for use in both the food and the nutraceuticals and cosmetic field.

## 1. Introduction

*Portulaca oleracea* L., commonly known as purslane, is a weed species belonging to the family *Portulacaceae*. The botanical Latin name of “small door” refers to the way they open the capsules. In the Middle Ages, it was known as “baqla hamqa” by the Arabs, which means “crazy plant” or “crazy”, most likely because of the way the branches extend over the ground without any control. Probably of Asian origin, it was used in ancient Egypt as a medicinal plant and in the Middle Ages, it spread both in Arab countries and in the Mediterranean Basin widely around Spain [1]. In recent years, it has been re-evaluated as a potential “new crop” by virtue of certain properties that distinguishes it as one of the best vegetable sources of omega-3 fatty acid (α-linolenic acid) and also possesses a high content of crude protein and water-soluble polysaccharides. These properties suggest a possible use of this species as a food and beyond in traditional medicine and cosmetics. Furthermore, *Portulaca oleracea* possesses a wide spectrum of pharmacological properties, as neuroprotective, anti-microbial, anti-diabetic, antioxidant, anti-inflammatory, anticancer and anti-ulcerogenic; for these reasons, it is traditionally widely used for therapeutic purposes [2]. In cosmetics, it is used for the development of new natural active principles for skincare, in fact, it presents an antioxidant capacity capable of preventing aging with a consequent reduction of wrinkles, and whitening capacity to eliminate stains caused by burns or excessive production of melanin [3]. A class of polyphenolic compounds present in purslane are flavonoids; these are biologically active and possess a wide range of pharmacological and antioxidant properties such as antibacterial, anti-viral, and anti-inflammatory [4]. In common purslane, the levels of flavonoids vary depending on the part of the plant; the highest levels of these compounds are present in the root, followed by the stem and leaves. Seven different types of flavonoids are present in this plant: kaempferol, myricetin, luteolin, apigenin, quercetin, genistein, and genistin [5]. In addition to flavonoids, the DOPA (3, 4-dihydroxyphenylalanine) compounds, dopamine and norepinephrine are present and belong to the family of alkaloids [6]. The content of dopamine and norepinephrine is greater in the leaves than stem and seeds, also, the amount of dopamine varies depending on the solvents used in the extraction process. The common purslane is also an excellent source of omega-3 fatty acids; this is particularly interesting given that, in general, they are found in oils and fatty fish, not plants. The omega-3 fatty acids play an important role in the enhancement of immune function, prevention and treatment of hypertension, coronary heart disease, and other inflammatory and autoimmune disorders. Of these omega 3 fatty acids, α-linolenic acid (ALA) is essential for normal growth, health promotion and disease prevention in humans. Moreover, the polysaccharides found in *Portulaca oleracea* have been reported as potential therapeutic agents for the treatment of diabetes mellitus by virtue of their ability to modulate the lipids and to decrease the amount of glucose present in the blood [7].

Current techniques of solid-liquid extraction essentially exploit diffusion and osmosis as the principles where it is possible to affect the reduction in extraction time and increase the yield. In fact, the current trend to achieve better results is to increase the temperature of the extractor system, or favor the contact of the extraction solvent several times with the solids to be extracted. A widely used extractive technique is maceration, where extraction takes place at room temperature and therefore there is no alteration of the thermolabile compounds. On the other hand, the extraction times are on average long because of the fact that the extraction takes place mainly by diffusion, so requires stirring of the system from time to time to promote the diffusion of the extracted compounds and avoid a localized supersaturation in the immediate vicinity of the surface of the solid to be extracted, which leads to an overall slowing of the extraction process. Ultrasonic extraction is a process that allows the extraction of the active principles contained in the plant by exploiting the mechanical action of ultrasound on plant walls, which break the cell walls to decrease the times of transfer of the active substances from the plant material to solvent. Yields obtained are very satisfactory in terms of active ingredients since it has an almost complete exhaustion of the vegetable. However, with ultrasound, it is not possible to make a selective extraction since it has the total leakage of all the molecules contained in the plant material, regardless of the affinity with the solvent used. A valid alternative to the existing extraction techniques is the solid liquid dynamic rapid extraction (RSLDE) using the Naviglio extractor, in this case the extraction is carried out for the generation of a negative pressure gradient from the inside to the outside of the solid matrix; therefore, it can be conducted at ambient or even sub-ambient temperatures. Experimental tests conducted to date have shown that most of the solid vegetable matrices, regardless of the degree of comminution, could be extracted using about twenty extractive cycles that are completed in about two hours. Furthermore, the extraction reproducibility was tested on the same matrix in terms of weighting and comparison experiments with other extraction techniques, which showed a higher recovery in favor of the Naviglio extractor, as well as a higher quality of the extract, and in no cases did it induce the alteration of heat-sensitive substances [8,9,10]. Therefore, the purpose of this work was to compare various solid-liquid extraction techniques to evaluate the most efficient technique for the extraction of biomolecules from leaves of purslane.

## 2. Materials and Methods

### 2.1. Materials

All reagents and solvents used in this study were of analytical reagent or HPLC grade and were purchased from Merck (Darmstadt, Germany). The raw leaves and sprouts of *Portulaca oleracea* were collected in the spontaneous state, in a region of southern Italy. Standards used to identify purslane antioxidant compounds were: caffeic acid; *p*-coumaric acid; scopoletin; ferulic acid; quercetin-3-*O*-rhamnoside; quercetin; apigenin; bergapten, all purchased from Sigma Aldrich (Milan, Italy), or were purified with an HPLC-UV/VIS working at 280 nm using the same chromatographic conditions described in the LC/MS/MS section from purslane leaves extracts. Fractions of the different compounds collected from HPLC were quickly freeze-dried, then dissolved in methanol at a concentration of 10 µg/mL and used for LC/MS/MS investigations. All the other reagents used were purchased from Sigma Aldrich (Milan, Italy).

### 2.2. Drying of the Purslane Leaves

The purslane leaves were removed from the plant and dried in a ventilated stove at 60 °C for 24 h. Comparing the weight of the leaves before and after drying, it was possible to calculate the percentage of moisture contained.

### 2.3. Extraction by Hot Maceration

Approximately 8 g of dried leaves of purslane were weighed. They were resuspended in 550 mL of distilled water, brought to a temperature of 70 °C and kept under stirring for 15 min. Finally, the extract was cooled down at room temperature, filtered and collected in a container. The extract was kept at −20 °C before the analyses.

### 2.4. Extraction by Naviglio Extractor

Approximately 8 g of dried leaves of purslane were weighed, included in a filter bag (Ø 50 μ) and inserted into the Naviglio extractor chamber of Lab. Model 500 cm^3^ capacity (Atlas Filtri Engineering, Limena, Padua, Italy). The extraction was conducted at a maximum pressure of 9 bars, the program was carried out in 30 cycles constituted by the alternation of a static phase (2 min) and a dynamic phase (12 s piston down; 12 s piston up for 5 cycles) and the extract was collected after 2 h using 550 mL of distilled water at room temperature. After extraction, the bag was emptied and squeezed to optimize the recovery and the extract was collected in a container.

### 2.5. Extraction by Maceration and Ultrasounds

About 8 g of dried leaves of purslane were weighed and resuspended in 550 mL of distilled water. The extractions were performed in an ultrasonic bath (Astrason Ultrasonic Clear, Farmingdale, NY, USA) with a working frequency of 33 KHz. The samples after sonication at room temperature for 15 min, were centrifuged to 4000 rpm, at 4 °C, filtered with filter paper and collected in a container.

### 2.6. Mix Extraction

As the name suggests, extraction was obtained using two methods in succession. First, 8 g of dried leaves of purslane, resuspended in 550 mL of distilled water were subjected to hot-maceration at 70 °C for 15 min under magnetic stirring and, subsequently, the Naviglio extractor was used. The latter phase had a duration of 1 h, performing 15 cycles of alternate static and dynamic phase (see above).

### 2.7. Dry Residue

To calculate the dry residue of the four extracts an exact aliquot of 10 mL from each extract was taken and placed in a beaker previously weighed. The extract was put at 60 °C in a ventilated stove, until to the evaporation of the solvent and the residue was weighed.

### 2.8. Liquid Chromatography-Tandem Mass Spectrometry System

An HPLC instrument equipped with a UV/Vis detector series 200 (Perkin Elmer, Shellton, CT, USA) was used for analysis; the wavelength for detection was 280 nm. A column Prodigy ODS3, 250 mm × 4.6 mm, particle size 5 µ, (Phenomenex, Torrance, CA, USA) was used for separation of compounds. Chromatographic conditions: flow = 0.8 mL/min; the program for gradient elution was the following: time 0 = 20% B; time 6 min = 20% B; time 16 min = 40% B; time 24 min = 50% B; time 32 min = 90% B; time 35 min = 90% B; time 38 min = 20%; split flow was 1:4; injection volume was 20 µL. An API 3000 (Applied Biosystems, Foster City, CA, USA) triple quadrupole mass spectrometer equipped with a Turbo Ion Spray source was used in the negative ion mode. Each sample was injected three times for the reproducibility.

### 2.9. Liquid Chromatography-Tandem Mass Spectrometry Analysis of Purslane Extract

Dried purslane leaves (3 g) were extracted at room temperature for 30 min with 30 mL of a mixture of water and methanol (30:70 *v*/*v*) by means of sonication. The extraction process was repeated three times for reproducibility. Next, the solutions were centrifuged at 6000 rpm for 5 min and then filtered through filter paper. Finally, the extract was analyzed by LC/MS/MS.

### 2.10. Folin-Ciocalteu Assay

For the analysis of Total Phenol Content (TPC) of purslane extracts, the Folin–Ciocalteu method [11] was applied with some modification of the previous method as indicated in the literature [12,13]. For 0.5 mL of each purslane extract solution diluted in methanol, 2.5 mL of diluted Folin–Ciocalteu reagent (1:10; *v*/*v*) was added; after 5 min, 2.0 mL of sodium carbonate (7.5% *w*/*v*) was added and the mixture let to react for 2 h at room temperature in the dark. Using 1 cm length cuvettes, the samples absorbances were measured at 760 nm. Finally, the total phenol content of the purslane extracts was reported in terms of mg/L of Gallic Acid Equivalents (GAE) as reported in References [12,13].

### 2.11. DPPH (2,2-diphenyl-1-picrylhydrazyl) Assay

The determination of the antioxidant activity was obtained by the DPPH assay using 2,2-diphenyl-1-picrylhydrazyl (DPPH Free Radical Scavenging Activity). The free radical scavenging activities of the extracts was determined as reported by Mishra et al. [14]. The DPPH showed a maximum absorption at 517 nm and the color changed from purple to yellow when the DPPH captured a hydrogen atom from an antioxidant. This reaction was stoichiometric with respect to the number of hydrogen atoms absorbed, thus the antioxidant effect was easily evaluated by following the decrease of UV absorption at 517 nm. The valuation was calculated as a percentage of absorbance decrease, also known as the percentage of inhibition. The antioxidant capacity was expressed as TEAC (Trolox Equivalent Antioxidant Capacity), which was defined as the amount of Trolox (mmol) necessary to obtain the same antioxidant activity. For samples, the Trolox calibration line was used to determine what corresponded in μmol TE/L as the percentage of inhibition.

### 2.12. Statistical Analysis

All data were expressed as mean ± SD of triplicate experiments (from three individual samples, each with three repetitions). Statistical comparison was carried out using the two-tailed student’s *t*-test. Statistical analyses were conducted using IBM SPSS Advanced Statistics (version 20; IBM, Armonk, NY, USA) considering *p* ≤ 0.05 as a significant difference.

## 3. Results and Discussion

### 3.1. Drying of the Purslane Leaves

Table 1 shows the determinations carried out on the purslane leaves before and after drying. The data obtained confirmed what was already known, that the percentage of moisture present in the leaves was generally always very high, averaging also around 90%.

### 3.2. Dry Residue

Table 2 shows the dry weight of the four types of the extract determined in 10 mL and 550 mL. In particular, it compares the dried residue (g/550 mL) obtained with four extraction methods with respect to the weight of the initial samples. From this table, it was possible to highlight that the mix method allowed us to obtain a greater quantity of extract than the other methods (3.06 g/550 mL).

### 3.3. Folin and Ciocalteu Assay

Table 3 shows the determination of polyphenols obtained from four extractions, in particular, the literature has shown that, using as solvent water with maceration method, the concentration of total polyphenols appears to be 142.8 ± 8.7 mg GAE/100 g fresh weight [15]. This value is consistent with the value calculated using the hot-maceration method (158.9 mg GAE/100 g fresh weight). For the extraction by the Naviglio extractor, the concentration of total polyphenols obtained was 67.4 mg GAE/100 g fresh weight. The extraction with ultrasounds allowed us to obtain a concentration of total polyphenols of 61.3 mg GAE/100 g of fresh weight, whereas the increased amount of polyphenols extracted was obtained through the mix method, which provided for two successive extractions, such as the hot-maceration at 70 °C for 15 min under magnetic stirring and, subsequently, the Naviglio extractor, shows the increased number of extracted polyphenols (237.8 mg GAE/100 g fresh weight).

Table 4 shows the results of the DPPH assay on the four types of extracts. This assay is not specific for polyphenols, but there is currently no other valid single method for measuring the antioxidant power of a compound. Consequently, the combination of the results of several assays based on different mechanisms and radicals should be used to reach a compromise that considers the final use of the results themselves. However, the DPPH assay is a quick, simple and inexpensive method that allows for the quantification of the reducing capacity of the substance under consideration whether it acts by hydrogen transfer or by the release of electrons, and was therefore used for our purposes. This test allowed us to have an idea of the antioxidant properties of the four types of extracts. Indeed, as can be seen, the Naviglio extractor (operating at room temperature) showed a high inhibition percentage (69.5%), whereas maceration with ultrasound showed an inhibition percentage of 65.4%, and the mix and maceration showed an inhibition percentage of 54% and 52%, respectively.

Furthermore, as can be seen from Table 3 and Table 4, the extracts obtained with the mix procedure had a Total Polyphenol Content (TPC) of 237.8 mg GAE/100 g of fresh weight, which was higher than the other methods, but exhibited a 54% inhibition rate. In fact, to compensate for this apparent contradiction, more methods should be used which when compared with each other, allow for a more accurate determination of antioxidant activity. However, it was sufficient to use only one method for our purposes, as the ultimate goal of this work was to determine the most efficient extraction method.

### 3.4. LC/MS/MS Characterization of Phenolic Compounds of Purslane Leaves

**Figure 1:**
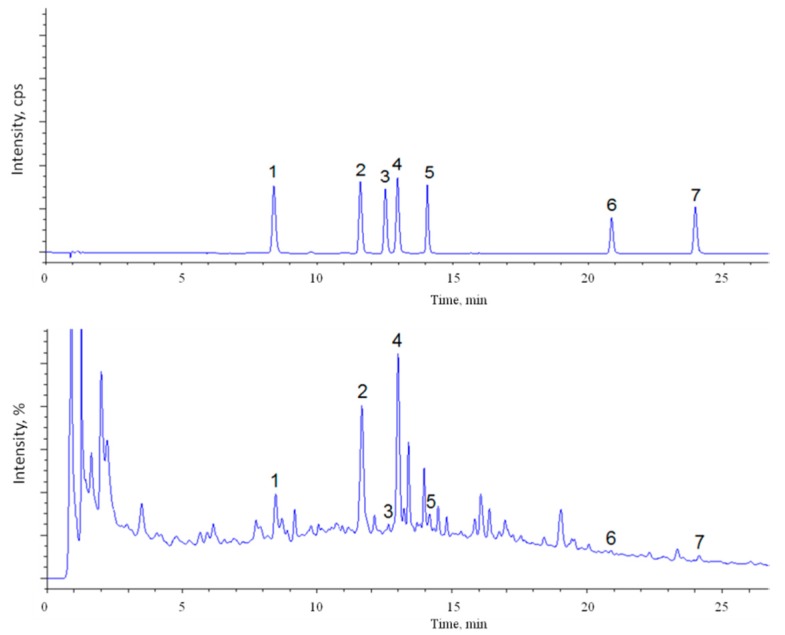
Total Ion Chromatogram (TIC) (**top**) and UV (**bottom**) at 280 nm of purslane leaves extract. The numbers correspond to the identified peaks: (1) caffeic acid; (2) *p*-coumaric acid; (3) scopoletin; (4) ferulic acid; (5) quercetin-3-*O*-rhamnoside; (6) quercetin; and (7) apigenin.

Purslane has been reported to possess potent pharmacological actions such as hepatotprotective, analgesic and anti-inflammatory, wound healing, neuropharmacological, bronchiodilatory, antidiabetic, antioxidant, antihypertensive, and many other biological actions [16]. Chemical constituents such as steroids, vitamins, minerals, fatty acids, alkaloids, saponins, etc. have been isolated from the plant [17,18]. For example, a recent research reported the simultaneous determination of the eight constituents present in *Portulaca oleracea* from different locations using the high-performance liquid chromatographic (HPLC) method. Results indicated that the contents of the eight components from different locations varied significantly, and therefore this data could be used to evaluate the quality of *Portulaca oleracea* [19]. The extensive literature survey revealed that *Portulaca oleracea* is an important medicinal plant with a diverse pharmacological spectrum. In particular, due to its high content of nutrients, especially antioxidants, purslane is also a very likely candidate as a useful cosmetic ingredient [20]. On the other hand, literature findings also reported that the concentration of phenolic compounds contained in the leaves was strongly dependent on the place where the plant was grown and that mature plants of *Portulaca oleracea* had higher total phenol content and antioxidant activities than plants at immature stages [15]. Therefore, in this work, phenolic compounds extracted by purslane leaves were identified by IDA acquisition and by comparison of the retention time of the standards. Successive leaf extracts were analyzed in Multiple Reaction Monitoring (MRM), which is known to allow an increase in the specificity of the detection system when more than one fragment was selected. In Figure 1, the Total Ion Chromatogram (TIC) and UV of an extract of purslane leaves are presented. Since most of the reported effects of purslane are due to its fresh juice or to its decoction, the aqueous extracts should be the most suitable [21,22]. However, further evaluations are needed to better characterize the extracts and evaluate their practical clinical applications to be used for health wellbeing.

## 4. Conclusions

This paper showed a comparison of solid-liquid extraction techniques on leaves of *Portulaca oleracea* L. to evaluate the most efficient process in terms of extracting bioactive molecules. From the experimental data on the comparison of the dry residues obtained with the different extraction techniques, it was found that the most efficient technique was the mix technique, combining two techniques, hot maceration and the use of the Naviglio extractor. In fact, with the mix extraction, a higher amount of polyphenols (237.8 mg GAE/ 00 g fresh weight) was obtained when compared to other techniques, ranging from 60–160 mg GAE/100 g fresh weight. However, the DPPH assay of the various extracts showed that mix extraction and maceration had a percentage inhibition of 54% and 52%, respectively, while extraction with the Naviglio extractor (operating at room temperature) had a high inhibition rate (around 70%). This led to the conclusion that the inhibition percentage was lower in high temperature extracts, which decreased antioxidant activity. Consequently, extracting with the Naviglio extractor represents a good compromise to obtain extracts with high antioxidant activity. In addition, this study was carried out a qualitative analysis of phenolic compounds present in the leaves of purslane examined by using LC/MS/MS by comparison with standard and literature data. The compounds were identified by comparing their molecular weight, fragmentation pattern, and retention time with those of standards using the Multiple Reaction Monitoring (MRM) mode, which, as is known, allows to increase the specificity of the detection system when more than one fragment is selected. In future, further studies will be carried out to quantitatively characterize the extract of greater interest. Therefore, the results of this paper, with the addition of further studies, will allow this wild plant with a large amount of biomolecules useful for beneficial effects on humans to be reevaluated. In addition, the richest extracts could be used in various fields such as the food, cosmetic and nutraceutical industries.

## Figures and Tables

**Table 1:** Determination of some values on the leaves of purslane before and after drying.

Purslane Leaves	Values
Initial weight of the leaves	400 g
Weight of the leaves after 24 h at 60 °C	44.3 g
Residual percentage	11%
Percentage of moisture	89%

**Table 2:** Dry residue in 10 mL and in 550 mL of four types of extracts.

Type of Extract	Dry Residue in 10 mL	Dry Residue in 550 mL
Maceration with ultrasound	0.0294 g	1.62 g
Maceration at 70 °C	0.0532 g	2.92 g
Naviglio extractor	0.0223 g	1.23 g
Mix extraction	0.0557 g	3.06 g

**Table 3:** Total Polyphenol Content (TPC) of the four types of extracts.

Samples	GAE mg/L	GAE mg/(8 g Dry Weight)	GAE mg/(100 g Fresh Weight)	% Polyphenols (8 g in 0.55 L)
Maceration with ultrasound	81.3	44.6	61.3	0.56
Maceration at 70 °C	210.4	115.5	158.9	1.44
Naviglio extractor	89.2	49	67.4	0.61
Mix extraction	314.4	172.9	237.8	2.16

**Table 4:** DPPH (2,2-diphenyl-1-picrylhydrazyl) assay on the four types of extracts.

Samples	Absorbance Samples	% Inhibition	DPPH Absorbance	μmol TE/L
Maceration with ultrasound	0.4102	65.4 ± 1.5	0.9011	52.8 ± 0.6
Maceration at 70 °C	0.5244	52.7 ± 0.8	0.9022	42.5 ± 0.8
Naviglio extractor	0.3737	69.5 ± 1.7	0.9011	56.1 ± 0.9
Mix extraction	0.5130	54.0 ± 0.9	0.9011	43.6 ± 0.7
